# Autoimmunity to tetraspanin-7 in type 1 diabetes

**DOI:** 10.1007/s00430-020-00674-2

**Published:** 2020-04-20

**Authors:** Kerry A. McLaughlin, Melissa A. Tombs, Michael R. Christie

**Affiliations:** 1 Oxford Centre for Diabetes, Endocrinology and Metabolism, University of Oxford, Oxford, UK; 2 School of Life Sciences, University of Lincoln, Lincoln, UK; 3 School of Life Sciences, Joseph Banks Laboratories, University of Lincoln, Lincoln, LN6 7DL UK

**Keywords:** Type 1 diabetes, Autoimmunity, Autoantibodies, Disease prediction

## Abstract

Type 1 diabetes is an autoimmune disease whereby components of insulin-secreting pancreatic beta cells are targeted by the adaptive immune system leading to the destruction of these cells and insulin deficiency. There is much interest in the development of antigen-specific immune intervention as an approach to prevent disease development in individuals identified as being at risk of disease. It is now recognised that there are multiple targets of the autoimmune response in type 1 diabetes, the most recently identified being a member of the tetraspanin family, tetraspanin-7. The heterogeneity of autoimmune responses to different target antigens complicates the assessment of diabetes risk by the detection of autoantibodies, as well as creating challenges for the design of strategies to intervene in the immune response to these autoantigens. This review describes the discovery of tetraspanin-7 as a target of autoantibodies in type 1 diabetes and how the detection of autoantibodies to the protein provides a valuable marker for future loss of pancreatic beta-cell function.

## Introduction

Type 1 diabetes is an autoimmune disease that results from the specific loss of insulin-secreting pancreatic beta cells leading to severe insulin deficiency and the requirement for insulin replacement therapy. The disease is associated with an inflammation within pancreatic islets, the clusters of cells within the pancreas where the beta cells are located, that is usually dominated by CD8^ +^ cytotoxic T-cells, with lower numbers of CD4^+^ helper T-cells, antibody producing B-cells and macrophages [1]. There is a genetic association of type 1 diabetes with HLA loci, specifically those associated with the presentation of antigens to CD4^+^ T-cells (HLA-DR3, HLA-DR4, HLA-DQ8), confirming an important role of the immune system in disease [2]. More than 90% of patients who develop type 1 diabetes have circulating autoantibodies to components of the affected tissue, the hallmark of autoimmune disease. Furthermore, these autoantibodies may appear many years before disease onset, providing valuable predictive markers for the future development of type 1 diabetes [3]. The availability of tools capable of identifying people at risk of developing type 1 diabetes, combined with the knowledge that the disease is caused by autoimmune destruction of beta cells, has led to a search for reagents capable of blocking autoimmune responses to the pancreatic beta cell to prevent disease developing in those identified as being at risk of disease. Indeed, immune intervention aimed at inactivating or depleting T-cells or B-cells has already shown promise in maintaining some residual beta-cell function during clinical trials involving patients with recent-onset type 1 diabetes, and more recently in those identified as being at high risk [4–7]. More effective tools for intervening in the immune response would be targeted very specifically at those immune responses directly involved in beta cell destruction, which requires identification of the targets of B-cells and T-cells that are activated in the disease. This review focuses on a member of the tetraspanin family, tetraspanin-7 (Tspan7) that has recently been discovered as a potentially important target of autoimmune responses in type 1 diabetes [8].

## Identification of islet cell autoantigens in type 1 diabetes

In 1982, Baekkeskov et al. [9] described studies in which proteins within isolated human islets were metabolically labelled with radiolabelled amino acid and extracts subjected to immunoprecipitation with antibodies in sera from patients with type 1 diabetes or from non-diabetic control individuals. In those experiments, distinct proteins of molecular weights 64 kDa and 38 kDa were immunoprecipitated by antibodies specifically found in sera from patients with type 1 diabetes. Since 1982, extensive research has been undertaken on the identification of targets of autoantibodies in type 1 diabetes, the development of assays for reliable detection of these autoantibodies and the design of strategies to use such assays in disease prediction. The 64 kDa protein originally described by Baekkeskov et al. was subsequently shown to represent two major targets of autoimmunity in type 1 diabetes: the 65 kDa isoform of glutamic acid decarboxylase (GAD65) and a tyrosine phosphatase-like protein, IA-2 [10, 11]. In addition, insulin and a zinc transporter, ZnT8, have also been demonstrated to be major targets of autoimmunity in the disease [12, 13]. Antibodies to all of these molecular targets are rarely detected in non-diabetic individuals. In people who develop diabetes, these autoantibodies appear months to years before the clinical onset of type 1 diabetes [14, 15], and often first appear within the first 5 years of life [16], making islet autoantibodies important markers for the identification of people at risk of developing type 1 diabetes, even in early childhood.

## Using autoantibodies to predict type 1 diabetes

A large number of studies have been performed to determine how the measurement of islet autoantibodies may be used to identify people at risk of developing type 1 diabetes. Having accurate markers for disease risk will be important for the introduction of disease prevention therapy. Most studies have been undertaken in families with type 1 diabetes, measuring islet autoantibodies in non-diabetic offspring or siblings of the proband with follow up for any later appearance of the disease. Some studies have followed family members from birth, providing valuable information on the time of appearance of individual islet autoantibodies and relationship to disease [14–17]. Such prospective studies on autoantibody appearance have demonstrated a progressive appearance of autoantibodies to insulin, GAD65, IA-2 and ZnT8, with insulin often the first antibody specificity to appear, often within the first five years of life [15–17]. There may be individuals where autoantibodies to GAD65 appear first at a later age [18]. Diversification of the immune response to multiple islet antigens appears to be critical for progression to type 1 diabetes; individuals who develop antibody responses to a single autoantigen without further diversification rarely develop type 1 diabetes, at least within the follow-up period of studies reported to date [19, 20]. Furthermore, no single antibody marker is able to provide optimum sensitivity and specificity for disease prediction. Instead, risk of progression to type 1 diabetes is best evaluated by assessing the number of individual antibody markers that are positive in a serum sample [19–21]. Currently, a combination of autoantibodies to insulin, GAD65, IA-2 and ZnT8 are often used to assess diabetes risk. The addition of new autoantibody markers may further improve our ability to predict type 1 diabetes, particularly when using a strategy where positivity for multiple antibodies provides the measure of risk.

## Identification of Glima 38 as tetraspanin-7

**Fig. 1:**
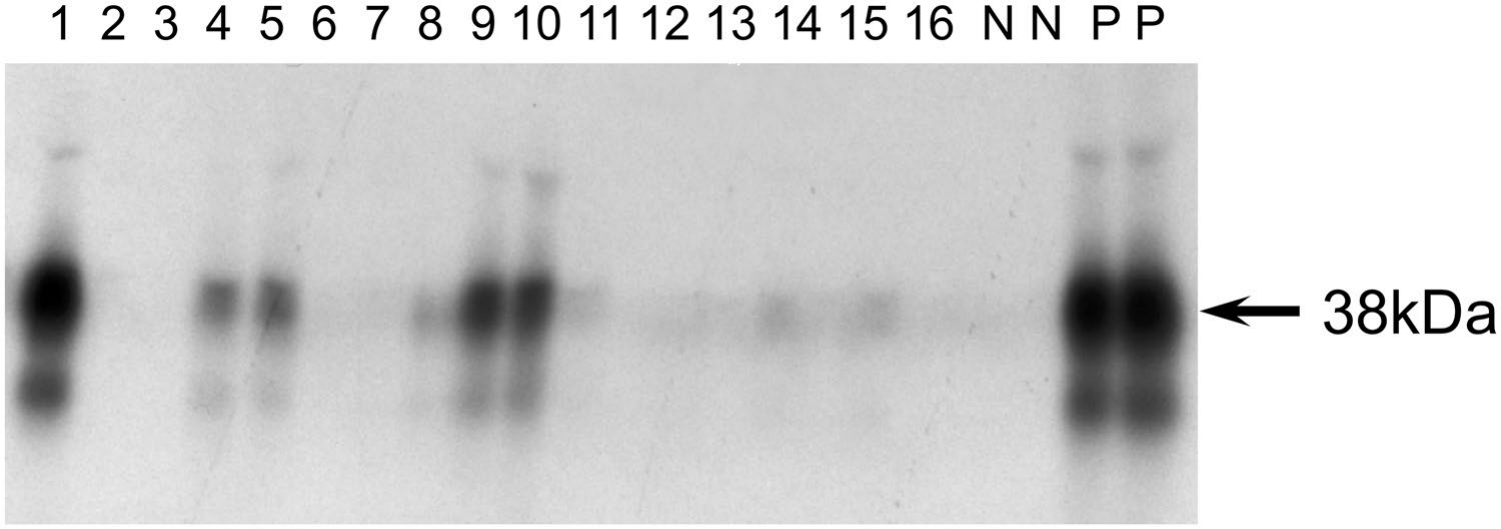
Immunoprecipitation of 38 kDa protein (Glima 38) by antibodies in sera from patients with type 1 diabetes. Proteins within the rat pancreatic beta-cell line, RIN 5AH, were radiolabelled with ^35^S methionine by incubation of the cells in medium containing ^35^S methionine. Proteins were extracted from labelled cells in Triton X-114 and subjected to temperature-induced phase separation to isolate amphiphilic membrane proteins (ref). Radiolabelled membrane proteins were incubated with sera from patients with type 1 diabetes overnight, immune complexes captured on protein A Sepharose, washed and separated by SDS PAGE. The figure shows the results of immunoprecipitation reactions with 16 patients with type 1 diabetes (1–16), two non-diabetic control samples (C), and two Glima 38 positive control samples. Diffuse 38 kDa bands, characteristic of heavily glycosylated membrane proteins such as the tetraspanin family, were specifically immunoprecipitated by antibodies in sera from patients with type 1 diabetes

Whilst the nature of the “64 k antigen” originally described by Baekkeskov et al. [9] was defined some years ago as two distinct proteins, the 65 kDa isoform of glutamate decarboxylase and IA-2 [10, 11], the identity of the 38 kDa component recognised by autoantibodies in type 1 diabetes has proved more elusive. Aanstoot et al. [22] performed a more extensive analysis of the nature of 38 kDa proteins immunoprecipitated from radiolabelled islets and cell lines by antibodies in type 1 diabetes together with an estimate of the proportion of patients positive for these antibodies. In that study, the authors showed that the 38 kDa protein partitioned into the detergent phase on phase separation in Triton-X114, a property characteristic of amphiphilic membrane proteins [23]. Furthermore, the protein resolved on SDS polyacrylamide gels as diffuse smears rather than sharp bands (Fig. 1), that was typical of heavily glycosylated membrane proteins. Indeed, deglycosylation of the protein with *N*-glycanase reduced the molecular weight of the protein to 22 kDa, indicating that the antigen has a high carbohydrate content. Substantial *N*-linked glycosylation of the antigen was further illustrated by a similar reduction in size when cells were incubated with the *N*-glycosylation inhibitor, tunicamycin, and by the ability of the protein to bind to the lectin, wheat germ agglutinin [24]. Since autoantibodies in patients’ sera were able to immunoprecipitate the non-glycosylated protein, the carbohydrate element appears irrelevant to antibody binding [22, 24]. Furthermore, the autoantibody target was found to be expressed in islet and neuronal cell lines, but not in a range of other transformed cell lines derived from other tissues, suggesting that, similar to other diabetes-associated autoantigens GAD65 and IA-2, the protein is specifically expressed in neuroendocrine tissues. Aanstoot et al. [22] dubbed the autoantigen Glima 38 (for Glycoslated Islet Membrane Antigen of 38 kDa).

The discovery of these specific characteristics of the novel autoantigen was expected to facilitate identification of the Glima 38 autoantigen [24], but a further 15 years were to elapse before the molecular identity of Glima 38 was finally defined. McLaughlin et al. took a proteomic approach to sequence fragments of proteins immunoprecipitated specifically by antibodies in sera from Glima 38-antibody-postive patients [8]. The authors demonstrated that high Glima 38 immunoreactivity could be detected in normal islets, brain, pituitary and lung and, for the purpose of Glima 38 purification and identification, amphiphilic membrane glycoproteins were isolated from extracts of brain and lung by Triton-X114 phase separation and wheat germ agglutinin affinity chromatography. These extracts were then further affinity purified using bead-conjugated antibodies isolated from sera of patients strongly positive or negative for Glima 38 antibodies. Eluates were separated by SDS polyacrylamide gel electrophoresis and 38 kDa bands treated with trypsin and analysed by liquid chromatography with tandem mass spectrometry (LC–MS/MS). The proteomic analysis identified three proteins present in both lung and brain extracts that were specifically eluted from the Glima 38 antibody-positive affinity beads but not from the negative control, and only one, tetraspanin-7, had the biochemical properties of Glima 38 described above. Tetraspanin-7 (Tspan7) was confirmed as a target of autoantibodies in type 1 diabetes by the ability to immunoprecipitate both the natural and recombinant protein expressed by cell lines by autoantibodies in patients’ sera [8].

Tspan7 is a 4-transmembrane domain protein with expression restricted to the central nervous system, to endocrine organs including the pancreatic islet and the pituitary, and to the lung [8]. Functional aspects of Tspan7 and the potential importance of the protein in neuronal development, viral infection and cancer are reviewed in this Special Issue [25]. There is evidence that the protein plays a role in vesicle trafficking in the cells of the nervous system and Tspan7 may regulate the surface expression of specific receptor molecules [25–27]; part of its function may be mediated by interaction with specific partner proteins [25, 27–29]. Mutations in Tspan7 have been associated with X-linked intellectual disability [25, 30–32] and impaired vesicle trafficking, cell surface receptor expression or dysregulated synapse development in the absence of functional Tspan7 may provide the molecular basis of the condition [25–27]. The role of Tspan7 in pancreatic islets is unknown, but one can speculate that the protein may also have a role in vesicle trafficking, given the secretory functions of cells in which the protein is expressed.

## Detection of autoantibodies to tetraspanin-7

A number of different assay formats are available for the detection of circulating antibodies to autoantigens associated with type 1 diabetes. Many of the research studies designed to quantify autoantibodies to GAD65, IA-2 and ZnT8 in type 1 diabetes have used radioligand binding assays in which radiolabelled antigen is generated by transcription and translation of the protein in vitro using appropriate plasmid constructs in the presence of radiolabelled amino acid. The translates are then incubated with serum samples, antibody-bound radiolabelled antigen captured on protein A Sepharose and quantified by scintillation counting [11]. Radioactivity immunoprecipitated then provides an indication of autoantibody levels. The protocol provides a relatively simple and versatile means to quantify diabetes-associated antibodies and the assay format has shown good reproducibility and performance in international diabetes autoantibody standardisation workshops [33–36]. Unfortunately, soon after the discovery of Tspan7 as an autoantibody target it was clear that radioligand binding assays with antigen transcribed and translated in vitro were not capable of detecting diabetes-associated Tspan7 antibodies effectively [8, 37].

**Fig. 2:**
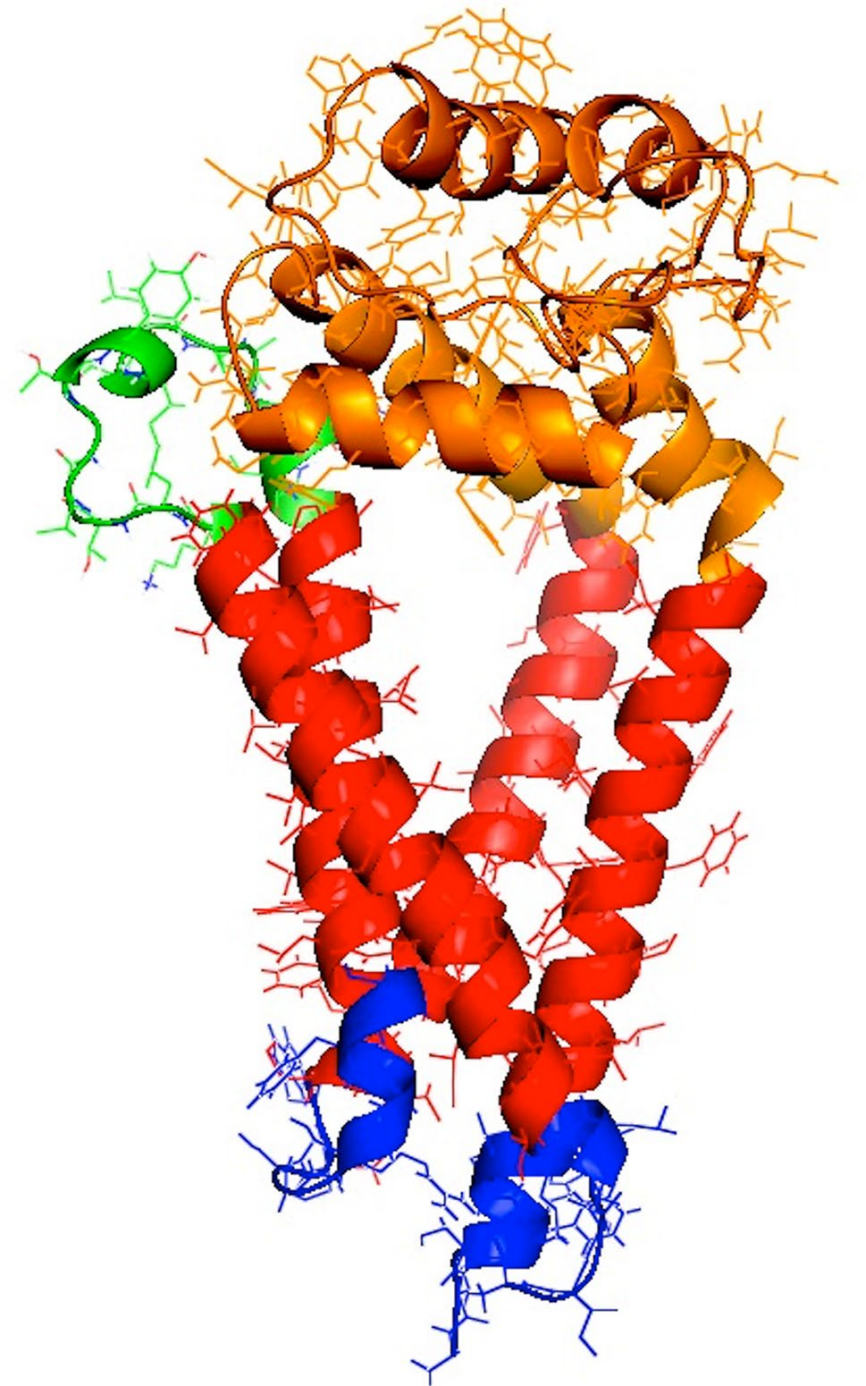
Model of structure of tetraspanin-7. The three-dimensional structure of Tspan7 was modelled by SWISS-MODEL [56] using the structure of CD81 as template. The figure shows the short and long ectodomains in green and orange, respectively, the four transmembrane domains arranged in a cone-like structure in red and the three short cytoplasmic domains arranged close together in blue. Epitopes for antibodies in type 1 diabetes are likely to lie within the cytoplasmic domains

The binding of autoantibodies to their target autoantigens in type 1 diabetes and other autoimmune disorders is very often highly dependent on the conformation of the target antigen, involving interaction with amino acids which may be quite far apart on the linear sequence of the protein, but which are located close together in the natural three-dimensional structure of the protein [38–40]. Production of the target antigen with appropriate three-dimensional structure is, therefore, critical for efficient autoantibody detection by immunoassay. The overall structure of tetraspanins, with four transmembrane domains, three short cytoplasmic domains and two glycosylated ectodomains (Fig. 2), presents a number of challenges for the development of assays for antibodies to the Tspan7 protein. Firstly, the hydrophobic nature of the protein, as a consequence of the four transmembrane domains, complicates its purification for use in immunoassays (for example ELISA or RIA) and may lead to high non-specific binding in various assay formats. Assays for antibodies to the transmembrane autoantigens IA-2 and ZnT8 usually have their transmembrane domains removed to avoid these issues [11, 13]. Secondly, if Tspan7 antibody epitopes involve amino acids from more than one of the three cytoplasmic domains, or from both ectodomains, then proper membrane insertion and alignment of the transmembrane domains may be required to form the epitope. This may not happen when Tspan7 is transcribed and translated in vitro and may explain the failure of Tspan7 antibody assays using protein expressed in this manner.

An alternative approach has, therefore, been taken to express Tspan7 in a form that can be bound by autoantibodies and easily detected and quantified. The assay format currently used is the luminescent immunoprecipitation system (LIPS) [8], in which the target antigen is expressed as a fusion protein with a luciferase tag that is incubated with test antibodies [41]. Antibody-bound luciferase-tagged antigen is captured on protein A Sepharose and quantified by luminometry after addition of a luciferase substrate. For Tspan7 antibody assays, the antigen is expressed as a recombinant protein after transfection of vector into mammalian cells, to increase chances of the protein folding naturally during protein synthesis. Because there is the potential for the interference of antibody binding to the antigen by the large luciferase tag, Tspan7 has been expressed as a fusion with nanoLuciferase, which is one of the smaller and brighter luciferases currently available.

Unlike radioligand binding assays with antigen transcribed in vitro, LIPS assays have proved capable of detecting of Tspan7 autoantibodies in type 1 diabetes [8]. Nevertheless, there have been issues with sensitivity and specificity of autoantibody detection by LIPS. Firstly, antibodies in a significant number of non-diabetic control samples have been shown to bind to the Tspan7-luciferase fusion protein, reducing the specificity of the assay [8, 37]. For these non-diabetic controls, natural or recombinant Tspan7 lacking a luciferase tag fails to block antibody binding, indicating that these antibodies are not specific to the Tspan7 molecule but rather bind a structure created by the fusion of Tspan7 with luciferase. To improve specificity, some Tspan7 autoantibody assays include a blocking step with untagged Tspan7, with effective inhibition of binding being an indication of Tspan7-specific binding [37]. Unfortunately, the higher specificity of this approach may be achieved at the expense of lower sensitivity.

The second problem observed with Tspan7 LIPS is that some samples in which Glima 38 antibodies were detected by immunoprecipitation assays with radiolabelled Glima 38 were negative in the LIPS, thereby reducing the sensitivity of the Tspan7 antibody assay [8]. Whilst it is possible that 38 kDa bands detected on autoradiographs after immunoprecipitation by antibodies in Type 1 diabetes sera may include proteins other than Tspan7, a more likely explanation is that some Tspan7 autoantibody epitopes may not be accessible on the recombinant antigen, either as a consequence of steric hindrance by the luciferase tag or because of issues with the conformation of Tspan7 when expressed as a recombinant protein; recombinant protein hyperexpression in mammalian cell expression systems may result in significant protein misfolding. Alternatively, it is known that several tetraspanins bind cholesterol [42] and structural analysis of the tetraspanin CD81 has revealed that the four transmembrane domains form a pocket in which cholesterol may bind. Incorporation of cholesterol into the CD81 structure dramatically alters the conformation of the tetraspanin [43]. If cholesterol-mediated conformational changes also occur for Tspan7, autoantibody binding may be disrupted because of alterations in the structure of the antibody epitope.

Further characterisation of antibody binding to chimeric or truncated constructs of Tspan7 has suggested that autoantibody epitopes lie predominantly within the first and third cytoplasmic domains, with potential antibody-contact residues identified at the C-terminal end of the protein by amino acid substitutions incorporated by site-directed mutagenesis [44, 45]. Autoantibody epitopes, therefore, lie within a relatively short (20-amino acid) region represented by at least two of the three cytoplasmic domains, providing further evidence of the importance of protein conformation in antibody binding. Establishing procedures to express or synthesise constructs that maintain the integrity of the autoantibody epitopes will be important for reliable detection of Tspan7 antibodies as markers of type 1 diabetes.

## Value of autoantibodies to tetraspanin-7 in diabetes prediction and prevention

The detection of autoantibodies to insulin, GAD65, IA-2 and ZnT8 has proved valuable for prediction and diagnosis of type 1 diabetes, has provided insights into disease pathogenesis and into the heterogeneity of immune responses that appear in individuals developing the disease. Identification of target autoantigens also opens up the possibility of specifically blocking immune responses to individual target antigens to prevent disease progression in those at risk, without the hazards associated with the administration of general immunosuppression. With the identification of a fifth major target in Tspan7, the question then arises as to the importance of Tspan7 autoimmunity to diabetes prediction and prevention. Because of the difficulties of establishing reliable Tspan7 antibody assays, there are currently very few studies addressing these questions.

Aanstoot et al. [22], using immunoprecipitation assays with radiolabelled cell extracts and assessing the presence of antibodies by detecting bands on autoradiographs, antibodies to Glima 38 (i.e. Tspan7) were found in only 19% of 86 recently diagnosed patients with type 1 diabetes aged 0.8–57 years. Antibodies to Glima 38 were found in a similar proportion (14%) of non-diabetic individuals who later developed type 1 diabetes and were present 3–74 months before disease onset. Glima 38 antibodies were not found in non-diabetic controls. Thus, Tspan7 autoimmunity can appear years before disease onset and may, therefore, be useful in disease prediction, but its value may be limited by the low proportion of patients positive for the antibodies.

Using a similar assay, with operators blinded to the identity of each sample, Winnock et al. [46] detected Glima 38 antibodies in a higher proportion, 38%, of patients with recent-onset type 1 diabetes (aged 0–39 years) and in 35% of non-diabetic individuals who later developed the disease. Similar to the Aanstoot study, Glima 38 antibodies were detected between 3 and 77 months before disease onset. None of 100 non-diabetic controls were positive in the assay. The differences in antibody frequencies between the two studies are likely due to problems in evaluating a positive result from the diffuse bands on autoradiographs, as the intensity of bands varies substantially between patients (Fig. 1). One might expect that introduction of quantifiable assays using recombinant antigen would improve the reproducibility and sensitivity, of Tspan7 autoantibody detection.

McLaughlin et al. [8], using a direct Tspan7 antibody LIPS, detected antibodies in 43% of 94 patients with recent-onset type 1 diabetes aged 12–63 years, and in 1 of 94 non-diabetic controls. The frequency of Tspan7 antibodies is higher in children than adults [47]. Walther et al. [37], using a similar LIPS assay but including a blocking step with untagged Tspan7, detected Tspan7 antibodies in 35% of 269 patients with recent-onset type 1 diabetes aged 7–13 years and positive for other islet autoantibodies, in 3.2% of 94 patients negative for islet autoantibodies and in 22% of 114 non-diabetic individuals considered at high risk of diabetes by having multiple antibodies to insulin, GAD65, IA-2 or ZnT8. Follow up of these high-risk individuals indicated that the addition of Tspan7 antibodies as a predictive marker resulted in a small but insignificant increase in the probability of diabetes progression [37].

It is now evident that diabetes is a heterogeneous disorder and tools that aid the categorisation of diabetes are needed to assist in providing the best treatments for individuals who develop the disease. For example, 10–15% of people initially diagnosed with type 2 diabetes are found to have evidence of beta-cell autoimmunity in the form of circulating islet autoantibodies and often progress to an insulin-requiring form of diabetes termed latent autoimmune diabetes in adults (LADA). Autoantibodies to GAD65 are commonly detected in LADA, whereas the frequency of those to insulin, IA-2 and ZnT8 are lower than found in acute-onset type 1 diabetes. LADA is generally considered to be a slow progressing form of autoimmune diabetes, perhaps associated with a less aggressive autoimmune response to pancreatic beta-cell antigens, with fewer patients having antibodies to multiple islet autoantigens.

Shi et al. [48] have recently examined the role of Tspan7 autoantibodies in type 1 diabetes and LADA in patients recruited in China. The prevalence of type 1 diabetes in Asia is lower than in the West and, in Asian patients who develop disease, antibodies to the established islet antigens such as IA-2 and ZnT8 are present at a lower frequency. For measurement of autoantibodies in the Chinese patients, the authors used a Tspan7 LIPS assay with a blocking step using untagged Tspan7 to confirm specificity, as developed by Walther et al. [37]. Antibodies to Tspan7 were detected in 26% of the Chinese type 1 diabetes patients, lower than the frequency of Tspan7 antibodies found in Caucasian patients with a similar assay [37]. Antibodies to IA-2 and ZnT8 were also lower in Chinese type 1 diabetes than in Caucasian patients, and the addition of Tspan7A antibodies did not significantly alter the proportion of patients identified with at least one islet autoantibody (from 82 to 84%). In contrast, measurement of Tspan7 antibodies was of value in predicting a decline in beta-cell function in Chinese patients with LADA. The addition of Tspan7 antibody analysis significantly increased the proportion of Chinese LADA patients with multiple antibodies and Tspan7 antibody positivity was associated with lower fasting and postprandial C-peptide levels, indicative of poorer pancreatic beta-cell function. Furthermore, the presence of Tspan7 antibodies predicted a faster decline in pancreatic beta-cell function in Chinese LADA patients, demonstrating that the novel autoantibody marker has value in predicting metabolic outcomes in specific diabetes patient groups.

## Tetraspanin-7 antibodies in other inflammatory disease

With the exception of insulin, all major targets of autoantibodies in type 1 diabetes are expressed outside of the insulin-producing beta cell, typically in cells of neuroendocrine origin. Furthermore, all autoantibody targets are intracellular, either by being located in insulin secretory vesicles (insulin) or by having autoantibody epitopes localised to the cytoplasmic domain of the molecule (GAD65, IA-2, ZnT8). Cytoplasmic location of autoantibody epitopes is also seen for Tspan7 [44, 45]. Because of this, it is unlikely that autoantibodies are directly involved in beta cell destruction themselves; instead, the selective targeting of beta cells by the immune system appears to be related to upregulation of MHC class I expression on the cell surface, leading to increased recognition by cytotoxic T cells [1]. Autoantibody-secreting B cells are likely to play a role in this process by processing and presenting antigen to both helper and cytotoxic T cells, facilitating their activation and function [49].

The expression of autoantigens in multiple organs may have significance in the development of other inflammatory diseases. For example, autoantibodies to GAD65, which is highly expressed in the brain, are described in several other autoimmune conditions, including Stiff Person Syndrome and polyendocrine autoimmune syndromes [50, 51]. Tspan7 is expressed in the brain, kidneys, liver and lung, as well as in pancreatic islets, and there is evidence that Tspan7 may be involved in an inflammatory disease affecting the lung, respiratory tract and kidney, granulomatosis with polyangiitis (GPA), formerly known as Wegener’s granulomatosis. Prior to the identification of tetraspanin-7 as a target of autoantibodies in type 1 diabetes, Thurner et al. determined the specificity of variable region immunoglobulin light and heavy chain genes from B lymphocytes that had been laser-dissected from germinal centres of Wegener’s granuloma. One of the putative targets identified was TM4SF2, the gene encoding tetraspanin-7 [52]. It is not yet clear whether circulating antibodies to Tspan7 are detected in patients with GPA. However, approximately 7% of patients with GPA co-present with diabetes mellitus; likewise, the presence of type 1 diabetes is associated with an increased familial risk of GPA [53, 54]. With the establishment of robust assays to measure antibodies to Tspan7, it may now be worthwhile investigating if they are indeed present in GPA patient populations.

## Concluding remarks

Tspan7 is established as an autoantigen in type 1 diabetes and may be considered a potential target for antigen-specific immune intervention to prevent disease in individuals identified as being at risk. Tspan7 is predicted to have the typical four transmembrane domain structure of the tetraspanin family, and the alignment of these domains appears important for creating epitopes for autoantibodies in disease, formed by the three short cytoplasmic loops linking the transmembrane domain. Difficulties in producing antigen with appropriate structure, together with the hydrophobic nature of the molecule, has hampered the development of reliable assays for antibodies to the antigen. Better understanding of the structure of autoantibody epitopes will be important for optimising Tspan7 antibody assays for prediction and autoimmune categorisation of type 1 diabetes, and potentially of other organ-specific inflammatory diseases such as GPA. Furthermore, because B cell and T cell epitopes frequently overlap [55], the cytoplasmic domains are also likely to harbour T cell determinants that could be targets of antigen-specific immunotherapy designed to inactivate autoreactive T cells in disease. The observation that autoimmunity in type 1 diabetes is directed to a relatively short regions of Tspan7 should assist in identifying T cell and B cell epitopes and will facilitate future studies to establish the importance of Tspan7 autoimmunity in the prediction and prevention of type 1 diabetes.
